# US College Students Are at Increased Risk for Serogroup B Meningococcal Disease

**DOI:** 10.1093/jpids/piz024

**Published:** 2019-05-11

**Authors:** Gary S Marshall, Amanda F Dempsey, Amit Srivastava, Raul E Isturiz

**Affiliations:** 1 Department of Pediatrics, University of Louisville, Louisville, Kentucky; 2 Department of Pediatrics, University of Colorado, Aurora, Colorado; 3 Pfizer Vaccines Medical Development, Scientific and Clinical Affairs, Collegeville, Pennsylvania

**Keywords:** college students, MenB, meningococcal disease, outbreak, vaccination

## Abstract

Publicly available surveillance data, Centers for Disease Control and Prevention reports, and other sources suggest that college students in the United States are at increased risk for meningococcus serogroup B (MenB) disease.

US surveillance data from 2015 to 2017 show that the incidence of invasive meningococcal disease (IMD) was greater among college students than among those not attending college; the average annual incidence of MenB disease was >5-fold higher among college students, and all college IMD outbreaks between 2011 and March 2019 were caused by MenB.

Invasive meningococcal disease (IMD) is life-threatening but vaccine-preventable. Meningococcal serogroups A, B, C, W, X, and Y account for almost all cases of IMD; meningococcus serogroup B (MenB) causes 38% of IMD in the United States and predominates (69% of cases) among persons aged 16 to 23 years [1].

In 2015, shortly after the first MenB vaccines—MenB-FHbp (Trumenba, bivalent rLP2086 [Pfizer Inc, Philadelphia, Pennsylvania]) and MenB-4C (Bexsero, 4CMenB [GlaxoSmithKline Vaccines, Srl, Siena, Italy])—were licensed in the United States, the Advisory Committee on Immunization Practices issued a category B recommendation (individual clinical decision making) for MenB vaccination of healthy persons aged 16 to 23 years, with a preferred age of administration of 16 to 18 years; persons at high risk, such as those with asplenia, complement deficiency, or laboratory or outbreak exposure, were included in a separate category A recommendation for persons aged ≥10 years [2]. Data available at the time failed to identify risk factors for MenB disease (beyond those covered under the category A recommendation) among otherwise healthy adolescents and young adults, which makes it challenging to guide parents regarding vaccination [2]. If college students are, in fact, at increased risk for MenB disease, the decision-making for MenB immunization of these patients would be affected significantly.

## METHODS

Meningococcal disease is a mandatory reportable condition in the United States. To reassess the risk of MenB disease among college students, we analyzed data compiled from the following surveillance systems: (1) the National Notifiable Diseases Surveillance System, which involves passive reporting of cases to the Centers for Disease Control and Prevention (CDC) from all US states and territories, (2) the Active Bacterial Core surveillance, an active population- and laboratory-based surveillance system that includes sentinel sites in 10 states and covers nearly 14% of the US population (approximately 44 million people) [3], and (3) Enhanced Meningococcal Disease Surveillance (EMDS). EMDS, initiated by the CDC in 2015, collects meningococcal isolates and key information on patients, including age, college attendance, human immunodeficiency virus status, vaccination history, and death. In 2016, EMDS included data from 45 states and 98% of the US population (approximately 317 million people) [1, 4]. It is important to note that data collected by these surveillance systems are different in terms of US population coverage, meningococcal serogroup identification, and outcomes, which likely accounts for the differences in case numbers reported herein. Higher-resolution data on cases of infection in college students, such as attendance at a 2- or 4-year institution, vocational school or community college, or residential or commuter school, are not available at this time. Data on campus outbreaks of meningococcal disease were gathered from CDC reports and other public sources. As an analysis of publicly available epidemiological data, this study was exempt from human subjects committee review.

## RESULTS

**Figure 1.**
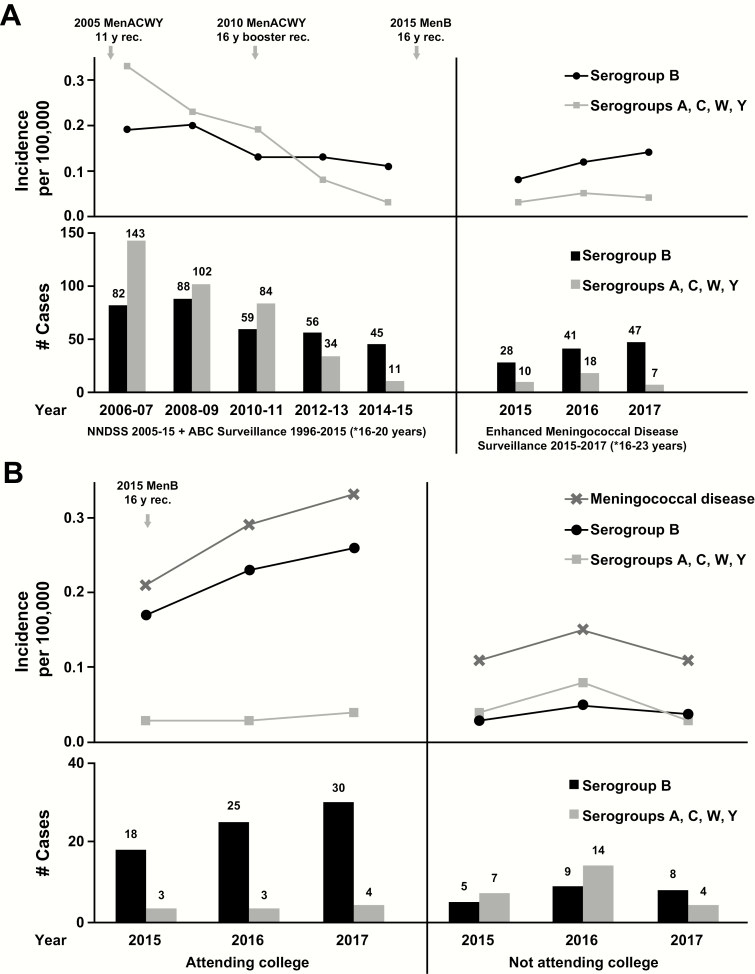
Meningococcal disease incidence and numbers of cases among individuals aged 16 to 24 years in the United States. (A) Meningococcal disease incidence and numbers of cases from 2006 to 2017. NNDSS and ABC surveillance data are for those aged 16 to 20 years, Enhanced Meningococcal Disease Surveillance data are for those aged 16 to 23 years. (B) Meningococcal disease incidence and numbers of cases according to serogroup and college attendance status. Data are from 2015, 2016, and 2017 Enhanced Meningococcal Surveillance System reports [1,4,5]. Abbreviations: ABC, Active Bacterial Core MenACWY, quadrivalent meningococcal conjugate vaccine (serogroups A, C, W, Y); MenB, meningococcal serogroup B vaccine; rec, recommendation; NNDSS, National Notifiable Diseases Surveillance System.

The incidence of IMD among 16- to 24-year-olds has declined since 2006 [3]. Around 2012, serogroup B became dominant over serogroups C, W, and Y combined [3]; this trend persisted through 2017 [1] (Figure 1A). Enhanced surveillance in 2015 to 2017 revealed a greater incidence of IMD (all serogroups) among 18- to 24-year-olds attending college than among those not attending college (Figure 1B), largely because of the increased incidence of MenB disease among those attending college [1]. From 2015 to 2017, the average annual incidence of MenB disease among college students (0.22 per 100 000) was >5 times that of those not attending college (0.04 per 100 000). In comparison with the 2009–2013 data that informed the 2015 recommendations [2], the current data show an increase in MenB disease burden among college students.

**Figure 2.**
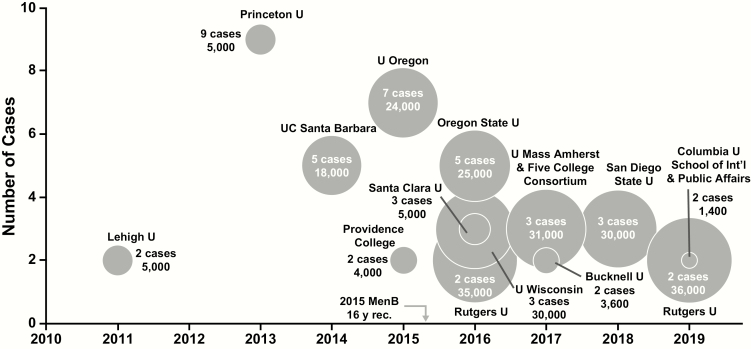
Meningococcal disease outbreaks on US college campuses between 2011 and March 2019. Bubble size corresponds to undergraduate enrollment. Data are from references 7–9 and announcements from university student health departments and local public health departments. Abbreviations: MenB, meningococcal serogroup B vaccine; rec, recommendation.

From 1994 to 2002, serogroup C caused the majority (57%) of organization-based IMD outbreaks (including those at colleges and universities) [8], but by 2009 to 2013, serogroup B predominated [7]. In fact, from 2011 through March 2019, MenB caused all US college outbreaks (Figure 2), which involved 13 campuses, 50 cases, and 2 deaths among an at-risk population of approximately 253 000 students.

## Discussion

The overall incidence of meningococcal disease in the United States has declined in the past few decades, and today it remains a rare disease. In that context, however, it is important to understand that college attendance seems to increase the risk of MenB disease, as it did the risk of disease caused by other meningococcal serogroups before the universal MenACWY vaccine recommendation [10]. The situation is roughly analogous to the increased risk of IMD that was seen in military recruits in the prevaccine era, the result of outbreaks that would occur when young adults from diverse geographic areas were brought together (under stress) into close living quarters [11]. The existing category A recommendation for MenB includes routine vaccination in outbreak situations, defined as 2 to 3 cases caused by the same serogroup in a specific population (such as a college campus) in <3 months [12]. Thus, the first 2 cases, and possibly the first 3, on a college campus would not be preventable under category A (because there is no “outbreak” until these infections have occurred). Important to note is that only 31.7% of MenB cases among 18- to 24-year-olds are outbreak associated [7 ]. Age-specific incidence data from 2014 to 2016 revealed that the relative risk of MenB disease among 18- to 24-year-old college students (3.54 [95% confidence interval (CI), 2.21–5.41]) was 3 times higher than that of non–college students, whereas the relative risk of serogroup C, W, or Y disease combined (0.56 [95% CI, 0.27–1.14]) was not increased. Although the risk for MenB disease among college students is distributed across the ages of 18 to 24 years, the age group at highest risk is 18- to 19-year-olds (3.10 [95% CI, 1.58–6.07]), which corresponds to the first year in college. These calculations include both sporadic and outbreak MenB cases; when MenB outbreak cases beyond the index cases were excluded, the relative risk of MenB disease among college students (2.76 [95% CI, 1.73–4.40]) decreased only marginally [7].

The 2 available MenB vaccines are compositionally distinct, have different vaccination schedules, and were tested differently for immune coverage of diverse MenB strains. Estimates using different methodologies predict that MenB-FHbp- and MenB-4C-elicited bactericidal antibodies will cover >91% of strains that circulate in the United States and/or Europe [13, 14]. After primary vaccination, MenB-FHbp demonstrated a composite seroprotection rate of 72.9%–85.7% against a panel of 4 diverse strains that were not matched to the vaccine components (across studies for 2- and 3-dose regimens) [15]. MenB-4C demonstrated a composite seroprotection rate of 63%–88% against 4 antigen-specific indicator strains that were closely matched to vaccine components (across studies for a 2-dose regimen) [16]. Other researchers found that the proportion of vaccine recipients with seroprotective antibody levels peaks 1 month after primary vaccination with either of these vaccines, and antibodies wane over 1 to 2 years [17–19]. Antibody persistence in those who receive either of the vaccines is difficult to generalize on the basis of the existing clinical studies. In 1 study, a single booster dose of either vaccine 4 years after the primary series elicited an immune response similar to the primary response and persisted for ≥2 years, which indicates robust memory [19]. These data suggest that vaccination at the age of 16 to 18 years is likely to provide protection against strains that college freshmen might encounter.

Only 14.5% of persons aged 17 years have received at least 1 dose of MenB vaccine, and likely even fewer complete the recommended vaccination series; in comparison, coverage rates for MenACWY are 83% for ≥1 dose in children aged 11 to 12 years and 44% for ≥2 doses in those aged 16 years (coverage rates among college students are not yet available) [20]. MenB disease prevention is likely to depend heavily on individual, rather than herd, immunity, because MenB vaccines do not seem to affect nasopharyngeal carriage among adolescents, and meningococcal carriage rates at outbreak and nonoutbreak college campuses are similar [21]. Although mass vaccination campaigns have been successful in stopping outbreaks [22], the containment approach presents logistical challenges (eg, rapidly and adequately immunizing students at risk), uncertainties (eg, outbreak duration and geographic footprint), and high costs [7]. It is worth noting that many colleges, including 3 of the largest public universities in Indiana, require MenB vaccine before matriculation [23]. At a minimum, information regarding the increased risk of MenB disease in college students should be available to all college-bound adolescents and their parents to inform the individual decision-making that is called for under the existing category B recommendation. Any discussion of routine (category A) vaccination of college-bound adolescents would need to include consideration of the cost of outbreaks, direct medical costs, the cost of vaccination, and the very low incidence of disease, among other factors.

## Notes

***Acknowledgments.*** This study was sponsored by Pfizer Inc. Pfizer employee-authors conformed fully to authorship requirements. Editorial/medical writing support (including development of the first manuscript draft) was provided by Jill E. Kolesar, PhD, at Complete Healthcare Communications, LLC (North Wales, Pennsylvania), a CHC Group company, and was funded by Pfizer Inc. We recognize Luis Jodar, PhD (head for Vaccines Medical Development and Scientific/Clinical Affairs at Pfizer Inc), for supporting this research.

***Author contributions.*** All authors contributed to the study conception and design, analysis and interpretation of data, and drafting and critical review of the manuscript and agreed to submit the manuscript for publication. All authors had access to the data, which were from publicly available surveillance systems.

***Financial support.*** This work was supported by Pfizer Inc.

***Potential conflicts of interest.*** G. S. M. has been an investigator on clinical trials funded by GlaxoSmithKline, Merck, Novartis, Pfizer, Sanofi Pasteur, and Seqirus, and he has received honoraria from these companies for service on advisory boards; he has also received honoraria from Pfizer and Sanofi for nonbranded presentations. A. F. D. serves as an advisor to Pfizer and on advisory boards for Pfizer, Merck, and Sanofi; she does not receive any research funding from these companies. A. S. and R. E. I. are employees of Pfizer Inc, and may hold stock or stock options.
